# Association between ultrasound BI-RADS signs and molecular typing of invasive breast cancer

**DOI:** 10.3389/fonc.2023.1110796

**Published:** 2023-05-17

**Authors:** Qiao-Hong Pan, Zheng-Pin Zhang, Liu-Yi Yan, Ning-Rui Jia, Xin-Yu Ren, Bei-Ke Wu, Yu-Bing Hao, Zhi-Fang Li

**Affiliations:** ^1^ Department of Ultrasound, Heping Hospital Affiliated to Changzhi Medical College, Changzhi, China; ^2^ School of Public Health, Shanxi Medical University, Taiyuan, China; ^3^ Department of Preventive Medicine, Changzhi Medical College, Changzhi, China

**Keywords:** breast neoplasms, molecular typing, ultrasonography, sonographic features, BI-RADS

## Abstract

**Objective:**

To explore the correlation between ultrasound images and molecular typing of invasive breast cancer, so as to analyze the predictive value of preoperative ultrasound for invasive breast cancer.

**Methods:**

302 invasive breast cancer patients were enrolled in Heping Hospital affiliated to Changzhi Medical College in Shanxi, China during 2020 to 2022. All patients accepted ultrasonic and pathological examination, and all pathological tissues received molecular typing with immunohistochemical (IHC) staining. The relevance between different molecular typings and ultrasonic image, pathology were evaluated.

**Results:**

Univariate analysis: among the four molecular typings, there were significant differences in tumor size, shape, margin, lymph node and histological grade (*P*<0.05). 1. Size: Luminal A tumor was smaller (69.4%), Basal -like type tumors are mostly larger (60.9%); 2. Shape: Basal-like type is more likely to show regular shape (45.7%); 3. Margin: Luminal A and Luminal B mostly are not circumscribed (79.6%, 74.8%), Basal -like type shows circumscribed(52.2%); 4. Lymph nodes: Luminal A type tends to be normal (87.8%), Luminal B type,Her-2+ type and Basal-like type tend to be abnormal (35.6%,36.4% and 39.1%). There was no significant difference in mass orientation, echo pattern, rear echo and calcification (*P*>0.05). Multivariate analysis: Basal-like breast cancer mostly showed regular shape, circumscribed margin and abnormal lymph nodes (*P*<0.05).

**Conclusion:**

There are differences in the ultrasound manifestations of different molecular typings of breast cancer, and ultrasound features can be used as a potential imaging index to provide important information for the precise diagnosis and treatment of breast cancer.

## Introduction

According to data released by the International Agency for Research on Cancer (IARC) in December 2020, breast cancer has become the most common malignant tumor that seriously threatens the health of women worldwide. Globally, there are 2.26 million new cases of breast cancer, accounting for 11.7% of the new cancer cases in the world (1, 2). In 2020, there was about 420,000 new cases of breast cancer, which constitutes 9.1% of new cancer cases in China. It caused about 117,000 deaths (3). In recent years, studies to identify benign and malignant breast disease by imaging are increasing, including use of artificial intelligence and radiomics (4, 5). Such studies intends to utilize the computer-aided diagnostic systems (CAD) to provide a decision-making for clinicians (6).

To facilitate communication and collaboration between clinicians and radiologists, the America College of Radiology (ACR) has proposed a unified terminology and diagnostic specification for evaluation of breast nodules, which is now widely cited clinically as the Breast Imaging Reporting and Data System 2013 Edition (7) (BI-RADS). Huang Qinghua et al. conducted an extensive study on the interconnection between AI and BIRADS ultrasound signs, which suggested that the use of AI and imaging histology can contribute much to the clinical practice, such as in reducing unnecessary punctures (8).

Additionally, patients with the same pathological classification and clinical stage may choose different treatment models due to different molecular subtypes, while the treatment sensitivity and clinical prognosis are also significantly different (9–12). Moreover, different molecular typings of breast cancer demonstrate different biological behaviors with diverse growth patterns, which inevitably lead to different ultrasound manifestations (13). Therefore, the identification of benign and malignant can no longer meet the clinical need for the treatment of breast diseases, whereas the imaging to predict molecular subtypes has been increasingly adopted, among which Color Doppler ultrasonography is a main imaging method to predict the molecular classification. This study aims intends to analyze the clinical data around diagnosis and treatment data of breast cancer patients, understand the characteristics of ultrasound images of breast cancer with different molecular typings, and determine the relationship between imaging and molecular typing. It will enhance the evidence base for accurate diagnosis and treatment of breast cancer, and for future research on artificial intelligence to facilitate the identification of molecular typing of breast cancer.

## Materials and methods

### Study participants

All female patients who had single side breast cancer, diagnosed by ultrasound examination within one month before surgery, and underwent surgical excision with complete pathological data were recruited. The included patients should be hospitalized in the Department of Breast Surgery, Heping Hospital Affiliated to Changzhi Medical College from May 2020 to October 2022. The patients who had breast surgery, neoadjuvant chemotherapy or radiotherapy, or pregnancy, or breast-feeding period were excluded. This study was approved by the Heping Hospital Medical Ethics Committee (No: RT2021142).

### Data collection

Demographic information and data of clinical management and ultrasound images were collected retrospectively, including age, body mass index (BMI), occupation, age of menarche, menopausal state, age of marriage, number of births, family history, histopathological grade and immunohistochemical results.

**Ultrasonic examination**: 1. Instruments and image acquisition methods: SΛMSUNG RS85, HITACHI ARIETTA 850, and SIEMENS OXANA 2 high-end color ultrasonic diagnostic instruments are used, respectively equipped with variable and high-frequency linear array probes LA4-18B, L5- 18, 18L6.Images were retrospectively analyzed by two doctors with more than 5 years of breast ultrasound diagnosis experience, using a blinded study design; that is, sonographers were not notified of the pathological diagnosis of patients during the analysis. And if there were differences of opinion, the final diagnosis would be made through consultation between the two. Acquire and store all ultrasound images with positive features and acquire dynamic images for later analysis if necessary. 2. Image processing: Ultrasound signs mainly refer to the “Breast Imaging Reporting and Data System 2013 Edition” (BI-RADS) published by the American College of Radiology (7). Ultrasound signs include the size of the mass, shape, orientation, margin, internal echogenic pattern, posterior echo, within the mass Calcification and axillary lymph nodes. According to the actual needs of clinical work, the calcification in the dictionary (no calcification, calcification within the mass, and calcification outside the mass) was adjusted to no calcification within the mass, micro calcification and coarse calcification.

### Histological and molecular tying examination

We used the postoperative pathology results to determine the histological tumor type and histologic grade. Tumor grading was classified as grade 1, grade 2 and grade 3. We considered grade 1 and 2 as low grade, while grade 3 as high grade. We tested estrogen receptor (ER), progesterone receptor (PR) and human epidermal growth factor receptor 2 (HER-2) expression levels by standard IHC staining. The pathological tissue classification refers to the requirements of Breast Tumors of WHO Breast Tumor Classification published in 2019 (14). Based on the St. Gallen consensus in 2013, breast cancer was divided into 4 molecular typings (15, 16): Luminal A like (LA), Luminal B like (LB), human epidermal growth factor receptor 2-positive (HER-2+), Basal-like, as shown in **Table 1**.

**Table 1:** the definition of molecular typing of breast cancer.

Subtype		ER	PR	HER-2	Ki-67
(LA)		(+)	(+)and ≥20%	(-)	<14%
(LB)	HER-2(+)	(+)	Any	(+)	Any
	HER-2(-)	(+)	(-)or <20%	(-)	≥14%
HER-2+		(-)	(-)	(+)	Any
Basal-like		(-)	(-)	(-)	Any

ER and PR staining: the positive rate is less than 1% as negative, and ≥1% as positive. HER-2 staining: a score of 0 or 1+ is judged as HER-2 negative, a score of 3+ is judged as HER-2 positive, and a score of 2+ is further performed for fluorescence *in situ* hybridization (FISH) test, if gene amplification was recorded as positive, and if no amplification was found, it was recorded as negative.

### Statistical analysis

All patients’ demographic and clinical characteristics were tested between different molecular typings. Chi-square test for categorical variables and one-way ANOVA for continuous variables. measurement data were tested for normality. Chi-square test or Fisher’s exact test was used for count data, and logistic regression was used for multivariate analysis. *P* value< 0.05 was considered statistically significant. Epidata3.0 software was used to develop a database and SPSS 26.0 software was used for data analysis.

## Results

### Population characteristics

Finally, a total of 302 subjects were enrolled in this study, We applied the formula of multiple regression to calculate the sample size, the minimum sample size was 270 cases all of whom were female, with an average age of 54.27 ± 10.24 years old. There was no significant difference in the population characteristics of different molecular typings, as shown in **Table 2**.

**Table 2:** Population characteristics of different molecular typing.

Parameters	LA (*n*=49)	LB (*n*=163)	HER-2+ (*n*=44)	Basal-like (*n*=46)	*x* ^2^ */F* value	*P*-value
Age (years ± SD)	56.88 ± 10.95	53.34 ± 10.16	53.41 ± 9.15	55.63 ± 10.43	1.895	0.130
Occupation (%)					6.219	0.101
Farmer	38 (77.6)	118 (72.4)	33 (75.0)	26 (56.5)		
Others	11 (22.4)	45 (27.6)	11 (25.0)	20 (43.5)		
Age of Menarche (years)	14.76 ± 1.54	14.61 ± 1.63	15.00 ± 1.83	14.98 ± 1.92	0.987	0.399
Menopause (%)					3.286	0.350
Yes	32 (65.3)	89 (54.6)	29 (65.9)	29 (63.0)		
No	17 (34.7)	74 (45.4)	15 (34.1)	17 (37.0)		
Age of Marriage (%)					1.255	0.740
≤23	32 (65.3)	115 (70.6)	29 (65.9)	29 (63.0)		
>23	17 (34.7)	48 (29.4)	15 (34.1)	17 (37.0)		
Fertility (%)					1.261	0.738
≤2	38 (77.6)	132 (81.0)	38 (86.4)	38 (82.6)		
>2	11 (22.4)	31 (19.0)	6 (13.6)	8 (17.4)		
Family History (%)					6.598	0.086
Yes	7 (14.3)	18 (11.0)	7 (15.9)	12 (26.1)		
No	42 (85.7)	145 (89.0)	37 (84.1)	34 (73.9)		
BMI ( $\bar{x}$ ± s)	24.91 ± 3.58	25.00 ± 3.18	24.23 ± 3.10	24.33 ± 3.43	0.978	0.403

Tests on the measurement data in the table found that age, BMI, and age at menarchemet normal distribution and equal variances, so groups were compared using the paired F-test.

### Pathological types and molecular typing

Among included patients 281 cases (93.1%) were non-specific type of invasive ductal carcinoma, whereas 21 cases (6.9%) were specific type of invasive breast cancer, among which 9 cases (3.0%) for invasive lobular carcinoma, 1 case (0.3%) for invasive tubular carcinoma, 4 cases (1.3%) for invasive mucinous carcinoma, 4 cases (1.3%) for invasive micropapillary carcinoma, and 3 cases (1.0%) for carcinoma with apocrine differentiation. More details are shown in **Table 3**.

**Table 3:** Histopathology of different molecular types of breast cancer [N(%)].

Molecular type	IDC	ILC	ITC	IMC	IMPC	AC	Total
LA	41(83.7)	2(4.1)	1(2.0)	2(4.1)	2(4.1)	1(2.0)	49(16.2)
LB	155(95.1)	4(2.5)	0(0.0)	2(1.2)	2(1.2)	0(0.0)	163(54.0)
HER-2+	43(97.7)	1(2.3)	0(0.0)	0(0.0)	0(0.0)	0(0.0)	44(14.6)
Basal-like	42(91.4)	2(4.3)	0(0.0)	0(0.0)	0(0.0)	2(4.3)	46(15.2)
Total	281(93.1)	9(3.0)	1(0.3)	4(1.3)	4(1.3)	3(1.0)	302(100)
*Fisher* value	18.091						
*P* value	0.096						

IDC, invasive ductal carcinoma; ILC, invasive lobular carcinoma; ITC, invasive tubular carcinoma; IMC, invasive mucinous carcinoma; IMPC, invasive micropapillary carcinoma; AC, carcinoma with apocrine differentiation.

### Histological grade and molecular typing

The numbers of patients sustained LA type, LB type HER-2+ type, and Basal-like type were 49 (16.2%), 163 (54.0%), 44 (14.6%), and 46 (15.2%), respectively.

Among them, LA, LB, and HER-2+ types had lower histological grades. LA type accounted for 95.9%, LB type accounted for 90.2%, HER-2+ type accounted for 86.4%, and Basal-like type accounted for more grade I-II tumors. High, grade III accounted for 45.7% (*P<*0.05), as shown in **Table 4**.

**Table 4:** Histologic grade of different molecular typings of breast cancer(%).

Molecular typing	Histological grade	
	I-II	III
LA	47(95.9)	2(4.1)
LB	147(90.2)	16(9.8)
HER2+	38(86.4)	6(13.6)
Basal-like1	25(54.3)	21(45.7)
*Fisher* value	33.628	
*P*-value	<0.001	

### Ultrasound imaging manifestations of different molecular typings

**Figure 1:**
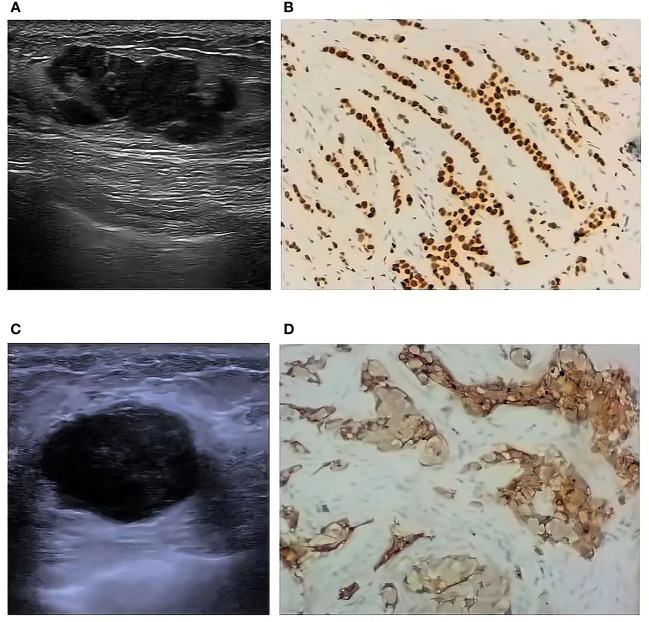
Ultrasound typical image and Immunohistochemical typical image of different molecular typing (10*10). Ultrasound typical image of a LA type breast cancer **(A)** and her Immunohistochemical typical image **(B)**; Ultrasound typical image of a Basal-like type breast cancer **(C)** and her Immunohistochemical typical image **(D)**.

The results of single factor analysis showed the tumor size, shape, margin, and ultrasound axillary lymph nodes were statistically different in different molecular typings of breast cancer (all *P*< 0.05). Tumor size: LA type tumor size is ≤ 2cm accounted for 69.4%, Basal-like masses were mostly larger (>2 cm, 60.9%); Tumor shape: Basal-like masses is more likely to show regular shape than LA, LB, and HER-2+ type (45.7% *VS* 16.3%, 11.0%, 15.9%); Mass margin: Most of Luminal types (LA and LB) were more not circumscribed (79.6% and 74.8%), and the Basal-like type mostly showed circumscribed margins (52.2%); Ultrasound lymph node manifestations: The structures of LA type ultrasound axillary lymph nodes tended to be normal (87.8%), and the structures of LB, Her-2+, and Basal-like lymph nodes tended to be abnormal (35.6%, 36.4%, and 39.1%), as shown in **Table 5**. Ultrasound typical image of a breast cancer was irregular in shape and not circumscribed, with no posterior echo attenuation (**Figure 1A**) and her Immunohistochemical molecular typing was LA type (**Figure 1B**); Ultrasound typical image of a breast cancer was of irregular shape, with circumscribed margins, and heterogeneous echo patterns (**Figure 1C**) and her Immunohistochemical molecular typing was Basal-like type (**Figure 1D**).

**Table 5:** Ultrasound imaging features of different typings of breast cancer (%).

Parameters	Status	LA (*n*=49)	LB (*n*=163)	HER2+ (*n*=44)	Basal-like (*n*=46)	*x* ^2^ value	*P-value*
Tumor size (cm)	≤2	34 (69.4)	84 (51.5)	20 (45.5)	18 (39.1)	9.738	0.021
	>2	15 (30.6)	79 (48.5)	24 (54.5)	28 (60.9)		
Shape	Regular	8 (16.3)	18 (11.0)	7 (15.9)	21 (45.7)	29.549	<0.001
	Irregular	41 (83.7)	145 (89.0)	37 (84.1)	25 (54.3)		
Orientation	Parallel	33 (67.3)	125 (76.7)	34 (77.3)	39 (84.8)	4.055	0.256
	Not parallel	16 (32.7)	38 (23.3)	10 (22.7)	7 (15.2)		
Margin	Circumscribed	10 (20.4)	41 (25.2)	13 (29.5)	24 (52.2)	14.887	0.002
	Not circumscribed	39 (79.6)	122 (74.8)	31 (70.5)	22 (47.8)		
Echo pattern	Hypoechoic	44 (89.8)	141 (86.5)	36 (81.8)	34 (73.9)	5.707	0.127
	Not hypoechoic	5 (10.2)	22 (13.5)	8 (18.2)	12 (26.1)		
Posterior echo	No/mixed change	22 (44.9)	80 (49.1)	20 (45.5)	23 (50.0)	12.258	0.056
	Enhancement	5 (10.2)	23 (14.1)	9 (20.4)	14 (30.4)		
	Shadowing	22 (44.9)	60 (36.8)	15 (34.1)	9 (19.6)		
Calcifications	No	32 (65.3)	78 (47.9)	23 (52.2)	28 (60.9)	6.265	0.394
	Microcalcification	12 (24.5)	59 (36.1)	16 (36.4)	13 (28.2)		
	coarse calcification	5 (10.2)	26 (16.0)	5 (11.4)	5 (10.9)		
Lymph nodes	abnormal	6 (12.2)	58 (35.6)	16 (36.4)	18 (39.1)	11.100	0.011
	normal	43 (87.8)	105 (64.4)	28 (63.6)	28 (60.9)		

### Ultrasound features of Basal-like breast cancer

**Figure 2:**
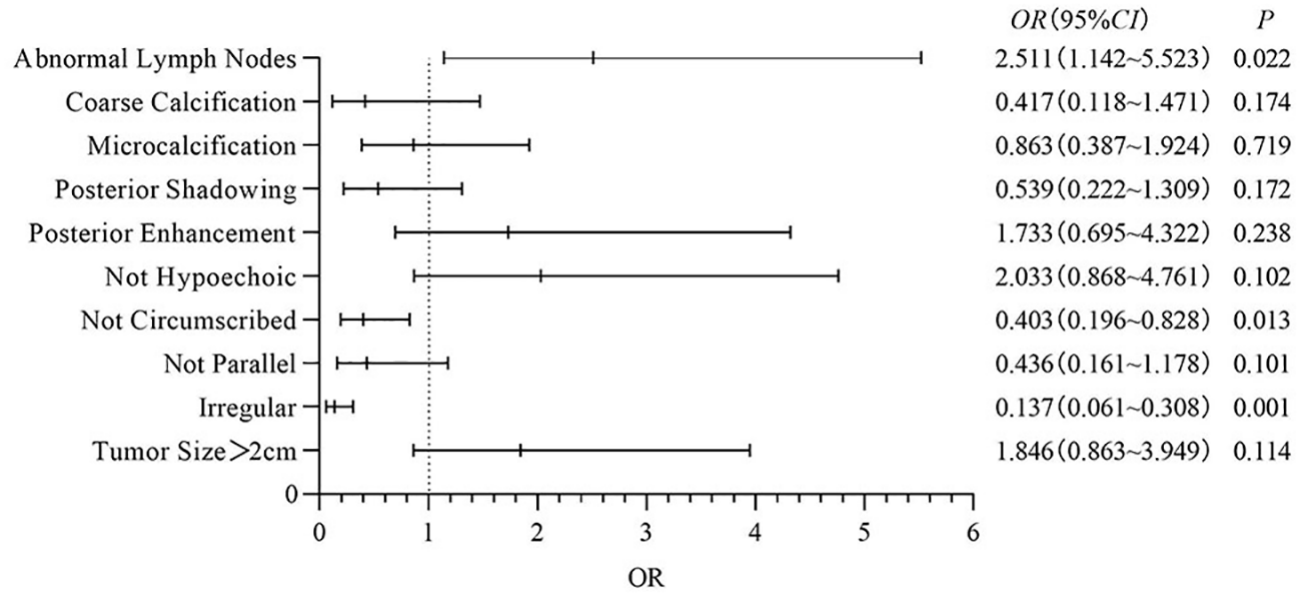
Ultrasound imagine features of Basal-like breast cancer.

Multivariate analysis: In this study, binary logistic regression analysis was performed on the ultrasound features of Basal-like breast cancer with poor clinical prognosis, and it was found that compared with other typings, irregular shape, not circumscribed margins and abnormal lymph nodes had a significant effect on Basal-like breast cancer (*OR*=0.137, 95%*CI*: 0.061~0.308, *P*<0.001; *OR*=0.403, 95%*CI*: 0.196~0.828, *P*=0.013; *OR*=2.511, 95%*CI*: 1.032~5.523, *P*=0.022). Ultrasound findings of different molecular typings breast cancer were statistically significant (*P<*0.05), other ultrasound features were not statistically significant (*P>*0.05), the results are shown in **Figure 2**.

## Discussion

At present, breast cancer has become a serious threat to women’s health and productive life. The identification of benign and malignant breast diseases by imaging means has achieved remarkable results, but the identification of benign and malignant breast diseases alone can hardly meet the clinical treatment of breast diseases. Existing research on breast cancer has delved into the field of genetics (17, 18). Breast cancer shows obvious heterogeneity due to different molecular typings, resulting in significant differences in its treatment and prognosis (19). Among them, endocrine therapy can significantly improve the prognosis of LA and LB types; molecular targeted therapy has a significant effect on patients with HER-2+ type (20); while Basal-like type currently lacks effective treatment methods, and has adverse prognosis (21). The purpose of this study was to explore the correlation between ultrasonography and molecular typing.

The results of this study showed no statistically significant differences in the pathological classification of each type of breast cancer (*P*=0.096), which may be analyzed as a result of bias in the results due to a small sample size.

There were statistically significant differences in the histological grades of various typings of breast cancer. Among the results, the Basal-like type grade III constituted 45.7%, which was significantly higher than that of other typings (*P*< 0.05). The similar findings was identified that the Basal-like type is more malignant and more aggressive (22).

Tumor size and lymph node metastasis have been considered to be important factors affecting the prognosis of breast cancer. Liu et al. (23) found that no lymphovascular invasion and no lymph node metastasis were associated with LA. The results of this study are similar. Compared with other typings, LA breast cancer has smaller lesions (≤2cm) and fewer abnormal lymph nodes, while Basal-like breast cancer has larger lesions (>2cm) and more abnormal lymph nodes. It shows that the prognosis of LA breast cancer is relatively good, while the prognosis of Basal-like breast cancer is relatively poor. In this study, the lymph nodes with abnormal ultrasound signs were all confirmed to be the lymph node metastasis of the primary tumor of breast cancer.

Lesions that directly invade the surrounding fat and fibrous tissue often appear irregular in shape on ultrasound, and the tumor tissue infiltrates and grows into the surrounding tissue, often showing not circumscribed margin. Basal-like breast cancer usually grows faster, and there are many tumor cells in the marginal area. It shows a pushing growth pattern, relatively less connective tissue reaction, and mostly shows regular shape and circumscribed margin on ultrasound (24). The results of this study are similar. The proportions of regular morphological and circumscribed margin masses in Basal-like breast cancer were 45.7% and 52.2%, respectively, which were significantly higher than those of other typings.

Abnormal metabolism and degeneration and necrosis of tumor cells can lead to calcification of malignant tumors. Microcalcification is a specific ultrasound sign for the diagnosis of breast cancer (25). Therefore, this study did not use the 2013 version of BI-RADS classification of intramass and extramass calcification, but divided them into no calcification, microcalcification, and coarse calcification in the mass. In this study, microcalcifications could not be used as a typing differential sign (*P*=0.394).An et al. believed that HER-2+ type showed more microcalcifications, and it was segmental and thin linear microcalcifications (26). The reason for the difference in the results may be that the age composition ratio of the research subjects is different. Moreover, An et al.’s study concluded that microcalcifications are the manifestations of mammography images. At present, there is still a gap between the performance of ultrasound detection of calcifications, especially microcalcifications and mammography.

Low-grade tumors are often accompanied by reactive hyperplasia of connective tissue, increased collagen fiber content, and posterior echo shadowing on ultrasound (27). In addition, studies have shown that there may be necrotic areas inside the lesions of Basal-like breast cancer, manifested as enhanced rear echoes, so theoretically there are differences in the rear echoes of each typing (28). But this study did not observe which was likely to be due to the result bias caused by the unbalanced composition ratio of the typings of the cases included in this study.

Hyperechoic lesions are uncommon in breast disease and are more likely to be benign (29). Most of the lesions in this study were hypoechoic, and the echo pattern was of limited value in distinguishing molecular typings. No statistical difference was observed in the echo pattern among the various typings, which is similar to the results of He et al. (30). The reported ratios of hyperechoic and mixed echoes in LB-type tumors were lower, while those in Basal-like tumors were higher (31). This may be due to the fact that there were too many patients with hypoechoic masses included in this study, and the proportion (255 cases, 84.4%) was too large.

The orientation of the mass is a unique feature of the mass on the ultrasound image, and more parallels indicate benignity, while non-parallel numbers indicate malignancy (7). In this study, the proportion of non-parallel growth tumors of each subtype ranged from 15.2% to 32.7%, and there was no statistical difference between the groups (*P*=0.256).

Because of the high degree of malignancy and strong invasiveness of Basal-like breast cancer, and the lack of effective treatment at present, the prognosis of Basal-like type is extremely poor. Therefore, this study compared the ultrasound signs of Basal-like breast cancer with other typings. Logistic regression analysis showed that Basal-like breast cancer was mostly characterized by regular shape, circumscribed margins and abnormal lymph nodes (*P*<0.05). This is supported by previous studies (32–34). This may be due to the high degree of malignancy of Basal-like breast cancer, relatively fast growth, tumor cell growth in the marginal zone, and less connective tissue response (24).

There are some limitations in this study. The retrospective observational study design is likely to induce selection bias (35). The small sample size, particularly each molecular typing might bias the study results. This study did not evaluate the consistency of different typings of ultrasound signs among different models of color Doppler ultrasound machines, but this might be aligned with the actual clinical practice.

## Conclusion

In this study, ultrasonographic presentation and clinicopathological data of 302 cases with different molecular typing of breast cancer were subjected to single-factor analysis and multi-factor analysis. It was found that among the four different molecular subtypes, there were statistical differences in mass size, shape, margins, lymph nodes and histological grading (*P*< 0.05), and no statistical differences in mass orientation, echogenic pattern, posterior echogenicity and calcification (*P* > 0.05). The most aggressive Basal-like type of breast cancer mostly showed regular shape, regular margins and abnormal lymph nodes (*P*< 0.05). In conclusion, different molecular typings of breast cancer have certain differences in their ultrasound features. Preoperative application of ultrasound to noninvasively predict the molecular typing of breast cancer is a new idea and method, which can provide a reference and basis for patients to choose a more accurate and individualized treatment plan.

## Data availability statement

The raw data supporting the conclusions of this article will be made available by the authors, without undue reservation.

## Ethics statement

The studies involving human participants were reviewed and approved by Medical Ethics Committee of Changzhi Medical College. Written informed consent for participation was not required for this study in accordance with the national legislation and the institutional requirements.

## Author contributions

Q-HP designed this study. Z-PZ wrote the manuscript, Z-PZ, L-YY and N-RJ participated in study selection and data extraction. Z-PZ, X-YR, B-KW and Y-BH performed statistical analysis. Q-HP and Z-FL reviewed the manuscript. All authors contributed to the article and approved the submitted version.
